# Demographic differences in complication and failure to rescue rates following lung transplantation

**DOI:** 10.1016/j.jhlto.2025.100440

**Published:** 2025-11-15

**Authors:** Boateng Kubi, Ruby Singh, Elsayed Elsafy, Orit Abrahim, Lucy Nam, Akash Premkumar, Stevens Bontemps, Dane C. Paneitz, Marlena Sabatino, Eriberto Michel, Asishana A. Osho

**Affiliations:** a Division of Cardiac Surgery, Massachusetts General Hospital, Harvard Medical School, Boston, MA, USA; b University of Massachusetts, Chan Medical School, Worcester, MA; c Department of Surgery, The Johns Hopkins Hospital, Baltimore, MD

**Keywords:** Transplantation, Disparities, Rescue, Complications, Mortality, Race, Lung

## Abstract

**Background:**

We evaluated the association between race/ethnicity and failure to rescue (FTR) following lung transplantation.

**Methods:**

We conducted a retrospective cohort study of 34,184 patients undergoing primary lung transplantation (2006–2024). Race/ethnicity was categorized as non-Hispanic White (81.3%), non-Hispanic Black (9.5%), and Hispanic (9.1%). The primary outcome was FTR. Logistic regression identified predictors of FTR, while Cox proportional hazards models assessed the association between race/ethnicity and outcomes.

**Results:**

Black patients experienced the highest rates of postoperative complications (26.3%) compared to non-Hispanic White (17.9%) and Hispanic patients (19.6%) (p < 0.001). Mortality rate was also highest among Black patients (6.8%) compared to White (5.0%) and Hispanic patients (4.8%) (p <0.001). However, race/ethnicity was not independently associated with FTR. Risk factors for FTR included dialysis at transplant (OR 2.05, 95% CI: 1.28–3.29, p=0.003), double lung transplant (OR 1.30, 95% CI: 1.09–1.56, p=0.004), and prolonged ischemic time (OR 1.05, 95% CI: 1.02–1.08, p=0.001).

**Conclusions:**

While Black patients experienced higher complication and mortality rates, race/ethnicity was not independently linked to FTR after lung transplantation.

## Introduction

Across surgical sub-specialties, differences in surgical outcomes by race/ethnicity have been well described.^1^, ^2^ These inequities contribute to an estimated 83,000 excess deaths annually in the United States and an added $250 billion in healthcare costs.^3^, ^4^ Solid organ transplantation—a complex, resource-intensive field—is not exempt from these disparities. Previous studies have consistently shown that racial and ethnic minority recipients experience worse postoperative outcomes and survival following kidney, liver, and heart transplantation.^5^, ^6^, ^7^, ^8^, ^9^ These racial/ethnic differences in outcomes have persisted despite novel allocation systems designed to reduce mortality and create equity in transplantation.^10^, ^11^ Their persistence likely reflects the complex, multifactorial nature of surgical inequity and the continued absence of actionable targets for intervention.

When assessing these mortality differences, available evidence suggests that they may not be reflective of the incidence of complications alone. Instead, they may arise from the inability to successfully intervene upon a complication before mortality occurs – a concept known as “failure to rescue” (FTR).^12^, ^13^ The term FTR, defined as mortality after a major complication, has become an increasingly accepted metric of quality and performance and has even been demonstrated to contribute to center-level differences in outcomes at transplant centers.^14^, ^15^ Within the surgical literature, hospital-level contributors to FTR such as low case volumes or resource constraints have received substantial attention.^16^, ^17^ Few studies have assessed patient-level factors that contribute to differences in FTR,^18^, ^19^ and perhaps to differences in postoperative outcomes by race/ethnicity.

In cardiothoracic surgery, the relationship between race/ethnicity and FTR remains understudied. Prior cohort analyses found Black race to be independently associated with increased rates of FTR after esophagectomy and congenital cardiac surgery.^20^, ^21^ In end-stage lung disease, Black patients face significantly worse waitlist survival compared to their White counterparts.^22^, ^23^ However, no prior studies have assessed the relationship between race/ethnicity and FTR after lung transplantation. The primary aim of this study, therefore, was to evaluate whether race/ethnicity is independently associated with FTR after lung transplantation in the United States. We hypothesized that minority patients would experience higher rates of complications, operative mortality, and FTR.

## Methods

### Study design and data source

We conducted a retrospective cohort study using data from the Organ Procurement and Transplantation Network (OPTN) Thoracic Registry. We included adult (≥18 years) recipients of primary single or double lung transplants performed between January 1, 2006, and March 31, 2024. Patients undergoing multiorgan transplants, re-transplantation, or with inadequate follow-up were excluded. The study was approved by the Mass General Brigham Institutional Review Board; individual consent was not required.

### Study variables

Recipient race/ethnicity, as reported during transplant listing, was categorized in our study as non-Hispanic White (White), non-Hispanic Black (Black), or Hispanic. Patients identifying as Asian, American Indian/Alaska Native, Native Hawaiian/Pacific Islander, or multiracial were excluded due to small sample sizes (<2% each). Recipient variables included age, sex, body mass index (BMI), underlying lung disease etiology, hospitalization or ICU admission at transplant, ventilator dependence, diabetes status, functional independence, renal function, type of transplant (single vs. double lung), lung allocation score (LAS), and insurance type. Transplant-specific variables included total waitlist time, ischemic time, and transplant era (2006–2010, 2011–2015, 2016–2024). Donor variables included age, sex, White race, BMI, creatinine, and diabetes status.

### Study outcomes

The primary outcome of interest was FTR, defined as operative mortality following a major postoperative complication. Operative mortality was calculated as a binary variable for mortality within 30 days of transplantation or during the index hospitalization, whichever was longer. Secondary outcomes included major complications such as airway dehiscence, acute kidney injury (AKI) requiring dialysis, stroke, acute rejection, and persistent extracorporeal membrane oxygenation (ECMO) support beyond 72 h. Composite FTR was calculated as the number of deaths among patients with any major complication divided by the total number of patients with complications. Complication-specific FTR was similarly calculated per complication type as summarized by the equation below:

$$\mathrm{FTR Rate}=\frac{\#\mathrm{of operative mortalities in patients experiencing at least one postoperative complication}\;}{\#\mathrm{of patients experiencing at least one postoperative complication}\;}$$

### Statistical analysis

Baseline characteristics were compared across racial/ethnic groups using Chi-square tests for categorical variables and Kruskal-Wallis tests for non-normally distributed continuous variables. Post hoc comparisons were performed using Dwass-Steel-Critchlow-Fligner procedures. Univariable and multivariable logistic regression models were used to identify predictors of FTR. Variables significant in univariable analysis (p < 0.5) were included in the multivariable model. All statistical analyses were performed using SAS version 9.4 (SAS Institute Inc., Cary, NC). A p-value <0.05 was considered statistically significant.

## Results

### Cohort characteristics

A total of 34,184 patients met inclusion criteria. Among these, 27,818 (81.3%) were White, 3261 (9.5%) Black, and 3105 (9.1%) Hispanic. The median age at transplantation was 61 years (IQR 53–66), with Black patients being the youngest group (median 56 years, IQR 48–62). Female recipients accounted for 40.1% of the cohort. Restrictive lung disease was the most common indication for transplant (52.0%). Black patients were more likely to be hospitalized, in the ICU, or on mechanical ventilation at the time of transplant (p < 0.001). They also had the longest median waitlist time at transplantation (57 days, IQR 17–174) and the highest proportion of public insurance coverage in our population (56.5%, p < 0.001). Baseline characteristics are demonstrated in Table 1.

**Table 1:** Transplant Recipient and Donor Characteristics Stratified by Race/Ethnicity

	Total (n=34,184)	White (n=27,818)	Black (n=3261)	Hispanic (n=3105)	p-value
Recipient Age, years	61 (53−66)	62 (55−67)	56 (48−62)	59 (49−65)	<.0001
Sex, Female	13722 (40.1%)	10631 (38.2%)	1777 (54.5%)	1314 (42.3%)	<.0001
Body mass index, kg/m^2	26.0 (22.4−29.2)	26.0 (22.3−29.1)	26.7 (23.0−29.7)	26.5 (23.2−29.5)	<.0001
Etiology of lung disease					
Obstructive	8666 (25.4%)	7905 (28.4%)	633 (19.4%)	128 (4.12%)	<.0001
Restrictive	17776 (52.0%)	13902 (50.0%)	1831 (56.2%)	2043 (65.8%)	<.0001
Cystic Fibrosis	3363 (9.84%)	3035 (10.9%)	118 (3.62)	210 (6.76%)	<.0001
Pulmonary Hypertension	1520 (4.45%)	1062 (3.82%)	262 (8.03%)	196 (6.31%)	<.0001
Other	2859 (8.36%)	1914 (6.88%)	417 (12.79%)	528 (17.0%)	<.0001
At transplantation					
Hospitalized	7618 (22.3%)	5773 (20.8%))	823 (25.2%)	1022 (32.9%)	<.0001
ICU	4176 (12.2%)	3096 (11.6%)	452 (13.9%)	628 (20.2%)	<.0001
Ventilator	1870 (5.47%)	1458 (5.24%)	163 (5.0%)	249 (8.02%)	<.0001
History of diabetes	6283 (18.4%)	4746 (17.1%)	706 (21.7%)	831 (26.8%)	<.0001
Independent of ADLs	7332 (21.8%)	6342 (23.2%)	537 (16.7%)	453 (14.9%)	<.0001
Creatinine	0.8 (0.7−1.0)	0.8 (0.7−1.0)	0.9 (0.7−1.1)	0.7 (0.6−0.9)	<.0001
Dialysis at time of transplant	132 (0.39%)	101 (0.36%)	13 (0.4%)	18 (0.6%)	0.18
Double lung transplant	24960 (73.0%)	19945 (71.7%)	2651 (81.3%)	2364 (76.1%)	<.0001
Total wait list time (d)	48.0 (14.0−154.0)	49.0 (14.0−154.0)	57.0 (17.0−174.0)	41.0 (13.0−137.5)	<.0001
Ischemic time (hr)	5.3 (4.3−6.6)	5.3 (4.3−6.6)	5.5 (4.3−6.8)	5.3 (4.2−6.4)	<.0001
Lung allocation score	40.9 (35.1−53.4)	39.8 (34.6−51.0)	43.0 (36.5−58.1)	48.0 (39.9−70.3)	<.0001
Public Insurance	18230 (53.3%)	14647 (52.7%)	1841 (56.5%)	1742 (56.1%)	<.0001
Era of transplantation					<.0001
2006−2010	6649(19.5%)	5700 (20.5%)	597 (18.3%)	352 (11.3%)	
2011−2015	8291 (24.3%)	7023 (25.3%)	734 (22.5%)	534 (17.2%)	
2016−2024	19244 (56.3%)	15095 (54.3%)	1930 (59.2%)	2219 (71.5%)	
Donor variables					
Age	34 (23−46)	33.0 (23.0−46.0)	34.0 (24.0−46.0)	35.0 (24.0−47.0)	0.007
Female Sex	13439 (39.3%)	10345 (37.2%)	1494 (45.8%)	1600 (51.5%)	<.0001
White race	20969 (61.3%)	17636 (63.4%)	1843 (56.5%)	1490 (48.0%)	<.0001
Body mass index	25.5 (22.6−29.3)	25.5 (22.5−29.2)	25.6 (22.6−29.4)	25.8 (22.7−29.4)	0.19
Creatinine	1.0 (0.7−1.4)	1.0 (0.7−1.4)	1.0 (0.7−1.4)	1.0 (0.7−1.6)	0.144
Diabetes	2744 (8.10%)	2197 (8.0%)	249 (7.7%)	298 (9.7%)	0.002

The median (interquartile range) is presented for all continuous data, and categorical data are presented as frequency (%). ICU = intensive care unit; ADL = activities of daily living

### Perioperative mortality and complications

Overall operative mortality was 5.2% (n = 1767). Black patients experienced the highest mortality rate (6.8%), compared to White (5.0%) and Hispanic (4.8%) patients (p<0.001), as shown in Table 2. Postoperative complications occurred in 26.3% of Black patients, 19.6% of Hispanic patients, and 17.9% of White patients (p < 0.001). There were no significant differences in the incidence of stroke or airway dehiscence by race/ethnicity. However, Black patients had the highest rates of acute rejection (8.8%), AKI requiring dialysis (12.5%), and prolonged ECMO support (14.6%) (p < 0.05 for all) (Table 3).

**Table 2:** Postoperative Outcomes and Composite Failure to Rescue Rate Stratified by Race/Ethnicity

Race/Ethnicity	Mortality	p-value	Complication Rate	p-value	Failure to Rescue Rate	p-value
		<.0001		<.0001		0.55
White	1396 (5.02%)		4980 (17.9%)		906 (18.2%)	
Black	221 (6.78%)		859 (26.3%)		168 (19.6%)	
Hispanic	150 (4.83%)		607 (19.6%)		117 (19.3%)

**Table 3:** Major Postoperative Complication Rates by Race/Ethnicity

Race/Ethnicity	Stroke	p-value	Acute Rejection	p-value	AKI requiring HD	p-value	Airway dehiscence	p-value	Persistent ECMO	p-value
		0.24		0.004		<.0001		0.65		<.0001
White	669 (2.4%)		2076 (7.5%)		1818 (6.6%)		413 (1.5%)		1212 (7.2%)	
Black	88 (2.7%)		287 (8.8%)		407 (12.5%)		54 (1.7%)		314 (14.6%)	
Hispanic	64 (2.1%)		207 (6.67%)		229 (7.4%)		51 (1.7%)		242 (10.1%)	

AKI = acute kidney injury; HD = hemodialysis; ECMO = extracorporeal membrane oxygenation

### Failure to rescue

As shown in Table 2, composite FTR rates did not significantly differ by race/ethnicity (White: 18.2%, Black: 19.6%, Hispanic: 19.3%; p = 0.55). Similarly, no racial differences were observed in FTR rates following stroke, acute rejection, or airway dehiscence. However, Black patients exhibited significantly higher FTR rates following AKI requiring dialysis (4.6% vs 2.3% for White patients) and persistent ECMO (3.8% vs 1.7% for White patients) (p < 0.001 for both) (Table 4).

**Table 4:** Failure To Rescue Rates by Individual Complication and Race/Ethnicity

Race/Ethnicity	Stroke	p-value	Acute Rejection	p-value	AKI requiring HD	p-value	Airway dehiscence	p-value	Persistent ECMO	p-value
		0.18		0.82		<0.001		0.81		<0.0001
White	162 (0.6%)		166 (0.6%)		648 (2.3%)		107 (0.39%)		280 (1.7%)	
Black	26 (0.8%)		17 (0.5%)		148 (4.6%)		15 (1.8%)		81 (3.8%)	
Hispanic	14 (0.5%)		19 (0.6%)		95 (3.1%)		12 (2.0%)		42 (1.8%)	

AKI = acute kidney injury; HD = hemodialysis; ECMO = extracorporeal membrane oxygenation

### Multivariable analysis

In adjusted logistic regression (Table 5), race/ethnicity was not independently associated with increased FTR risk. Factors associated with higher odds of FTR included dialysis at the time of transplant (OR 2.05, 95% CI 1.28–3.29, p = 0.003), double lung transplant (OR 1.30, 95% CI 1.09–1.56, p = 0.004), and prolonged ischemic time (OR 1.05, 95% CI 1.02–1.08, p = 0.001). Protective factors included younger age (18–45 years: OR 0.66, 95% CI 0.53–0.82, p = 0.001), functional independence at transplant (OR 0.74, 95% CI 0.62–0.89, p = 0.002), transplant in the modern era (2016–2021: OR 0.71, 95% CI 0.59–0.85, p < 0.001), and White donor race (OR 0.78, 95% CI 0.69–0.89, p = 0.0002).

**Table 5:** Univariable and Multivariable Analysis of Factors Associated with Failure to Rescue

	Univariable			Multivariable		
	OR	95% CI	p-value	OR	95% CI	p-value
Recipient variables						
Age						
18−45	0.68	0.56, 0.83	0.001	0.66	0.53, 0.82	0.001
46−64	0.87	0.76, 1.0	0.43	0.84	0.72, 0.97	0.66
65−79	Ref			Ref		
Female Sex	0.96	0.85, 1.09	0.29	0.93	0.80, 1.08	0.35
Race						
White	Ref			Ref		
Black	1.09	0.91, 1.31	0.6	1.18	0.97, 1.42	0.28
Hispanic	1.07	0.87, 1.33	0.81	1.11	0.89, 1.39	0.86
Body mass index	1	0.99, 1.02	0.54	1	0.98, 1.01	0.59
Etiology of lung disease						
Obstructive	0.95	0.81, 1.12	0.54	0.81	0.60, 1.10	0.17
Restrictive	1.18	1.04, 1.34	0.01	1.06	0.91, 1.2	0.46
Cystic Fibrosis	0.82	0.65, 1.03	0.08	1.04	0.78, 1.38	0.8
Pulmonary Hypertension	1.1	0.87, 1.38	0.43	1.23	0.91, 1.67	0.17
Other	0.79	0.64, 0.97	0.02	0.86	0.68, 1.09	0.22
At transplantation						
Hospitalized	0.91	0.80, 1.05	0.19	0.97	0.83, 1.14	0.74
ICU	1.01	0.87, 1.18	0.89	1.13	0.94, 1.35	0.2
Ventilator	1.04	0.85, 1.28	0.68	1.08	0.86, 1.35	0.54
History of diabetes	1.02	0.87, 1.19	0.85	1.02	0.86, 1.23	0.8
Independent of ADLs	0.76	0.64, 0.91	0.003	0.74	0.62, 0.89	0.002
Creatinine	1.15	1.02, 1.28	0.02	1.08	0.96, 1.22	0.2
Dialysis at time of transplant	2.19	1.40, 3.43	0.001	2.05	1.28, 3.29	0.003
Double lung transplant	1.06	0.91, 1.24	0.45	1.3	1.09, 1.56	0.004
Total wait list time (d)	1	1.00, 1.00	0.07	1	1.00, 1.00	0.16
Ischemic time (hr)	1.02	1.00, 1.04	0.06	1.05	1.02, 1.08	<0.001
Lung allocation score	1	1.00, 1.00	0.98	1	1.00, 1.01	0.64
Public Insurance	1.02	0.90, 1.16	0.71	0.94	0.81, 1.10	0.45
Era of transplantation						
2006−2010	Ref			Ref		
2011−2015	0.92	0.75, 1.12	0.66	0.87	0.71, 1.06	0.73
2016−2024	0.78	0.66, 0.92	0.002	0.71	0.59, 0.85	<0.0001
Donor variables						
Age	1.01	1.00, 1.01	0.0002	1.01	1.00, 1.01	0.0001
Female Sex	1.06	0.94, 1.2	0.35	0.98	0.85, 1.14	0.82
White race	0.79	0.70, 0.90	0.0002	0.78	0.69, 0.89	0.0002
Body mass index	0.99	0.98, 1.00	0.04	0.99	0.98, 1.00	0.048
Creatinine	1.03	0.99, 1.06	0.18	1.02	0.97, 1.06	0.48
Diabetes	1.12	0.90, 1.40	0.31	1.01	0.78, 1.31	0.96

ICU = intensive care unit; ADL = activities of daily living; Ref = reference

## Discussion

In this large, national cohort study of lung transplant recipients, we found that while Black patients experienced the highest rates of postoperative complications and operative mortality, there was no independent association between race/ethnicity and failure to rescue after adjustment for clinical factors. Notably, Black patients were more likely to present to transplant with a higher burden of acute and chronic illness, as evidenced by baseline comorbidities, likelihood of hospitalization, and likelihood of ICU admission at the time of transplant. Taken together, these findings suggest that differential preoperative conditions drive the observed inequity in postoperative mortality rather than disparate rescue efforts.

The mechanism behind this preoperative condition for black patients is complex and has been well documented in the literature describing the social determinants of health.^24^, ^25^, ^26^, ^27^, ^28^ One lever that should be highlighted is the previously described observation that minority populations face significant barriers to accessing healthcare.^25^, ^26^ Minority communities have decreased access to transportation and a lower insured population rate, yielding unaddressed health conditions that lead to higher comorbidity burden at the time of transplant.^25^, ^26^, ^27^ Specifically, Black patients were found to experience decreased health care utilization and disability compensation while having increased waiting list death rates. ^28^ Furthermore, current literature has demonstrated that current race-based spirometry equations lead to discriminatory diagnostic decisions, further delaying transplant access.^28^

FTR has emerged as a key surgical quality metric and reflects a system’s ability to detect, respond to, and mitigate serious complications.^14^, ^15^ Despite the increasing utilization of FTR, the patient-level factors that contribute to it are poorly elucidated. Prior studies have primarily focused on institutional factors such as staffing ratios, case volumes, and technological resources.^29^, ^30^ In the emergency general surgery literature, advanced age and lack of insurance are the two patient factors found to contribute to increased FTR.^18^, ^19^ Less is known about the influence of patient-level factors, particularly race/ethnicity, on FTR.

In our cohort, Black and Hispanic patients had higher crude FTR rates after specific complications such as AKI requiring dialysis and prolonged ECMO. However, these differences did not persist in multivariable models, suggesting again that preoperative illness severity and transplant-related factors accounted for the observed disparities. Conversely, factors that were protective of FTR in our analysis included young age, baseline independence, contemporary transplantation era, and having a White donor.

Despite being the youngest cohort, Black patients did not derive the expected survival benefit of younger age, which may indicate a blunted physiologic reserve or unmeasured social determinants of health that adversely affected their recovery, as mentioned previously.^31^ Conversely, White transplant recipients had the lowest postoperative complication rate in our cohort, despite being the oldest group with a median of 62 years. Interventions targeting modifiable preoperative risk factors, particularly in socially vulnerable populations, may be important in reducing these disparities. Potential approaches include standardized peri- and post-transplant care pathways that account for comorbidity burden and broader, social-worker–informed risk assessments to surface unmet needs.

Our study has a few limitations. First, as a retrospective registry analysis, we were limited to the data elements collected in the OPTN database and could not account for center-level variables such as staffing, protocols, or quality improvement efforts. For example, acute rejection diagnosis depends on biopsy practice patterns, which may vary by center, and thus is susceptible to misclassification bias. Second, although we used a validated set of major complications, causal attribution between specific complications and mortality cannot be confirmed. Additionally, the available variables may not capture all clinically meaningful events and that unmeasured complications could influence FTR estimates. Third, important social determinants of health—such as housing instability, access to post-discharge care, and health literacy—were not captured but likely impact both complication risk and recovery. Fourth, racial/ethnic groups with small sample sizes were excluded, which may limit generalizability. Finally, our study was not designed to assess specific changes in transplant care over time, especially those intended to prioritize equity and eliminate disparities.

## Conclusions

In this large cohort of lung transplant recipients, Black patients experienced the highest rates of postoperative complications and mortality. However, race/ethnicity was not independently associated with failure to rescue, suggesting that higher early mortality among minority patients is driven primarily by increased baseline risk. Interventions aimed at optimizing pre-transplant health and addressing social determinants of risk may be critical to improving equity in lung transplantation outcomes.

## Disclosures

The authors report no proprietary or commercial interest in any product mentioned or concept discussed in this article.

## Funding

None.

## CRediT authorship contribution statement

Boateng Kubi, Eriberto Michel, and Asishana A. Osho conceived the study. Boateng Kubi, Ruby Singh, Orit Abrahim, Lucy Nam, and Akash Premkumar curated the data. Formal analysis was conducted by Boateng Kubi and Ruby Singh. Boateng Kubi, Ruby Singh, Orit Abrahim, and Marlena Sabatino led the investigation. Methodology was developed by Boateng Kubi and Asishana A. Osho. Project administration and resources were provided by Asishana A. Osho. Software support was provided by Boateng Kubi and Ruby Singh. Supervision was provided by Asishana A. Osho and Dane C. Paneitz. Validation and visualization were performed by Boateng Kubi with input from Asishana A. Osho. Boateng Kubi drafted the original manuscript. All authors contributed to critical review and editing of the final manuscript, including Ruby Singh, Orit Abrahim, Dane Paneitz, Lucy Nam, Akash Premkumar, Marlena Sabatino, Eriberto Michel, and Asishana A. Osho.

## Declaration of Competing Interest

The authors declare that they have no known competing financial interests or personal relationships that could have appeared to influence the work reported in this paper.
